# Perspectives of Policymakers on the Introduction and Scale-Up of HIV Self-Testing and Implication for National HIV Programming in Ghana

**DOI:** 10.3389/fpubh.2021.694836

**Published:** 2021-09-21

**Authors:** Henry Nagai, Henry Tagoe, Waimar Tun, Edward Adiibokah, Augustine Ankomah, Yussif Ahmed Abdul Rahman, Stephen Ayisi Addo, Stephen Kyeremeh Atuahene, Emmanuel Essandoh, Mark Kowalski

**Affiliations:** ^1^JSI Research and Training Institute, Inc., Accra, Ghana; ^2^Population Council, Accra, Ghana; ^3^Population Council, Washington, DC, United States; ^4^National AIDS/STI Control Programme, Accra, Ghana; ^5^Ghana AIDS Commission, Accra, Ghana; ^6^United States Agency for International Development, Accra, Ghana; ^7^JSI Research and Training Institute, Inc., Washington, DC, United States

**Keywords:** policymakers, men who have sex with men, female sex workers, Ghana, HIV self-testing

## Abstract

**Background:** HIV self-testing (HIVST) has the potential to greatly increase HIV testing uptake, particularly among key populations (KPs) at higher risk for HIV. Studies have shown high acceptability and feasibility of HIVST among various target populations globally. However, less is known about the perspectives of policymakers, who are critical to the success of HIVST implementation. Their views on barriers to the introduction and scale-up of self-testing are critical to understand in order for HIVST to become part of the national HIV guidelines. We sought to understand policymakers' perspectives of challenges and facilitators to the introduction of HIVST at the client and structural levels.

**Method:** Key informant interviews (KIIs) were conducted with national and regional level policymakers involved in the HIV response. Twenty policymakers were purposively selected from Greater Accra (capital) and Brong-Ahafo (outlying) regions. Qualitative content analysis was used to arrive at the results after the verbatim transcripts were coded.

**Results:** Client-level challenges included lack of pre-test counseling, the need for confirmatory testing if reactive, potential for poor linkage to care and treatment, and client-level facilitator from policy makers' perspectives included increase testing modality that would increase testing uptake. Structural-level challenges mentioned by policymakers were lack of a national policy and implementation guidelines on HIVST, cost of HIVST kits, supply chain management of HIVST commodities, data monitoring and reporting of positive cases. The structural-level appeal of HIVST to policymakers were the reduced burden on health system and HIVST's contribution to achieving testing targets. Despite the challenges mentioned, policymakers unanimously favored and called for the introduction of HIVST in Ghana.

**Conclusions:** Findings indicate that a non-conventional HIV testing strategy such as HIVST is highly acceptable to policymakers. However, successful introduction of HIVST hinges on having national guidelines in place and stakeholder consultations to address various individual and structural -level implementation issues.

## Background

Closing the HIV testing gap and reaching the first 95 of the WHO's 95-95-95 targets is critical to the success of the global and national HIV response (1). The HIV epidemic in Ghana is characterized as a mix of a low-level generalized epidemic with a prevalence of 1.6% in the general population (2) but a high prevalence among female sex workers (FSWs) (4.6%) and men who have sex with men (MSM) (18.1%) (3–5). HIV testing remains low with only an estimated 55% of people living HIV who know their status (6). Testing is less than optimal among persons at high risk of HIV infection across the country. In a national survey among FSWs in 2019, only 56.5% tested for HIV in the last 12 months preceding the survey (5). The situation is far worse among MSM as only 26.6% of MSM tested and received the results in the last 12 months preceding the 2017 Ghana Men Study II (4). The low uptake of HIV testing services (HTS) in the country, particularly among those at high risk for HIV results in high numbers of undiagnosed HIV infection. This situation presents a major challenge toward achieving epidemic control in the country.

Conventional facility-based and provider-assisted HTS have inherent barriers to universal access to testing and treatment. These barriers include stigma, negative provider attitudes and discrimination, limited confidentiality, and limited convenience (7–9). The potential of HIV self-testing (HIVST) to increase HIV testing uptake as an entry point to the HIV/AIDS care continuum, especially among the highly stigmatized and hard-to-reach populations, are well-documented (7, 10–17). Convenience, confidentiality, and privacy are highly influential in the acceptability and utilization of HIVST (13, 18–20). These advantages of HISVT would help accelerate the progress toward reaching the WHO/UNAIDS's 90-90-90 targets and effectively link people to HIV prevention and treatment services including antiretroviral therapy (ART), condoms and other prevention services, prevention of mother-to-child transmission (PMTCT), and pre-exposure prophylaxis (PrEP).

The level of acceptability and utilization of HIVST is high as identified in implementation research and randomized controlled trials globally (12, 14–16, 21–26). Studies in African countries have also reported high levels of willingness to use HIVST as well as actual HIVST use when made available to the population. A qualitative exploratory study in two regions in Ghana among FSWs and MSM recruited by outreach workers and peer educators of community-based organizations, the majority of participants expressed willingness to use HIVST kit (27). The evidence in the literature demonstrates high acceptability and uptake of HIVST across different populations. However, national policies for HIVST are still missing for many countries, including Ghana. For example, as of June 2020, there were still 16 countries in Africa with no policies explicitly allowing HIVST, although many of these countries are in the phase of developing such policies (28). National policies around HIVST are not only important for increasing access to HTS, but it is also critical for ensuring quality of products and safe and ethical usage.

Policymakers or policy influencers who drive national policy agenda about HIVST are central for the adoption of HIVST as national HIV testing strategy. The National Strategic Plan 2016-2020 for HIV in Ghana proposes the introduction of HIVST as an important component in the approach of getting KPs to test for HIV and be linked to care (29). The Strategic Plan mentions that HIVST is not yet approved in Ghana and that there is need to pilot HIVST before a policy directive can be made. Understanding the perspectives of policymakers on the potential barriers and facilitators is critical to obtain government approval for the strategy and develop country-specific HIVST guidelines. This article interrogates how policymakers in Ghana perceive the introduction of HIVST in Ghana, specifically with respect to challenges and facilitators at the client and structural levels. This understanding will help drive policy strategy and HIVST implementation as the country introduces HIVST as a testing strategy to increase HIV testing access and uptake.

## Methods

Key informant interviews were conducted in September-October 2017 with 20 national and regional policymakers in the area of HIV and AIDS policy and program implementation in Greater Accra (GA) and Brong Ahafo (BA) regions, which represents two of the then ten regions of Ghana. Greater Accra and Brong Ahafo regions were selected to represent the southern and northern zones, respectively, with Greater Accra being the most urbanized and cosmopolitan region in the country. The Brong Ahafo region lies in the middle belt of the country and is considered a transitional zone attracting populations from both northern and southern sections of the country. After the completion of the study, Brong Ahafo region was divided into three separate regions—Bono, Bono East, and Ahafo. Most study participants came from what is now considered the Bono region. Participants were purposefully selected from Ghana Health Service, National AIDS/STI Control Program (NACP), Ghana AIDS Commission (GAC), and Regional/District HIV Coordinators from Greater Accra and Brong-Ahafo regions. These are institutions whose activities directly determine and influence policy related to HIV testing. Participants had to be a national or regional director or manager working with any of these nationally established HIV and AIDS bodies.

Interview guides were developed by the authors and were field-tested to ensure that questions were appropriate for the intended respondents. Interviews explored potential facilitators and barriers to developing an official HIVST guidelines for Ghana, attitudes and perceptions regarding who should have access to HIVST kits and how it should be provided, impact of HIVST introduction on health systems, policy considerations surrounding HIVST introduction, cost and financing of HIVST, and service provision considerations, and commodity security and supply issues. Interviews were conducted by trained qualitative researchers in English over a period of 8 weeks starting November 2017, digitally recorded with permission from participant, and transcribed verbatim.

### Data Analysis

Five out of 20 transcripts were sampled across the different stratum (2 national and 3 regional) and given to the team of researchers (HT, EA, and WT) to read and develop codes. The codes generated were discussed in relation to the study objectives and the research questions that underpinned the study and consensus was reached among the team. The categories and sub-categories emerging from the transcripts were finalized into codebook with clear definitions. The codebook and database with transcripts were entered into a computer-assisted qualitative analysis software package (QSR NVivo 11). Two trained qualitative research assistants coded the transcript in the QSR NVivo 11. The research team (HT, EA, and WT) processed the coded data by running different queries along the main categories (individual and structural challenges and facilitators) using a qualitative content analysis approach (30, 31).

### Ethical Consideration

The Population Council Institutional Review Broad in New York and the Ghana Health Service Ethical Review Committee, Accra approved the study. All participants gave written informed consent before the interview. For confidentiality, we do not report institutional affiliations, but assigned a unique code to each policymaker to reflect national or regional level.

## Results

The 20 sampled policymakers consisted of 6 national and 14 (seven from each of the two selected regions) regional level policymakers. The designation of the national level policymakers were Director, Deputy Director of Clinical Care, and Director and Deputy Director of Administration. At the regional level, the Regional HIV Coordinator, Monitoring and Evaluation Officer, Regional and Data Manager were interviewed. The results section is divided into the two broad categories of client-level and structural-level challenges and facilitators to the introduction of HIVST in Ghana. The client level barriers were issues such as challenges related to pre-test counseling, confirmatory testing, linkage to care and treatment, and cost of HIVST kits, and client level facilitator was the increased option for testing, especially one that provides greater privacy, confidentiality, convenience with reduced stigma. The structural level challenges were the lack of national policy framework and implementation guidelines, and cost that would have implications for the large-scale implementation of HIVST. Structural level facilitators included reduced burden on the health system and potential to attain testing targets.

### Client-Level Challenges and Facilitators

#### Challenge 1: Lack of Pre-test Counseling

A concern expressed by many of the policymakers was that HIVST would not allow for sufficient pre-test counseling as required by the national HIV testing guidelines and as standardly practiced during provider-assisted testing. They were concerned that without pre-test counseling, clients would not understand the implications of their test results and not know how to seek further services. A few even mentioned that it may lead to negative outcomes such as suicidal ideations. They expressed this concern particularly for those who test for HIV for the first time.

“*…somebody who does not understand HIV issue and the testing issue well will eventually do the test, if you are not counseled, the results may scare you and you may not know what to do”* (National policymaker #04)

“*…it is going to cause a lot of if not suicidal tendencies, there is going to be a lot of suicides reports especially among the adolescence.”* (National policymaker #02)

#### Challenge 2: Need for Confirmatory Testing

Although a minority, a few policymakers expressed concern that individuals who use HIVST and obtain a reactive test result with the HIVST kit would not access confirmatory testing at an HIV testing site by healthcare providers, as required by the national HIV testing algorithm. They felt that KPs, in particular, would not want to present themselves at a public testing facility for confirmation of test result due to fears of stigma.

“*One key thing that I am also worried about is the fact that this is a primary test and there's the need to confirm it. One of the factors for self-test is the fact that the person doesn't want to be seen or done by somebody else. …It is because I don't want people to know I am a female sex worker; I do my test at home quietly and go. So, what is the assurance that people would move in for the confirmation test?”* (Regional policymaker BA #02)

“*[A person] may not going for a confirmation, she sits with it and dies with it and probably will not even go for medications.”* (National policymaker #01)

#### Challenge 3: Potential for Poor Linkage to Care and Treatment

Many policymakers expressed concerns about HIVST users not being linked to care and treatment. They pointed out that the absence of counseling with HIVST may be a hindrance for onward linkage to care and treatment.

“*If you are even positive, it may also delay linking you asking for care… you can seek care early, but you may also seek care late because you didn't receive adequate counseling, you did it on your own, you feeling ok the results is telling you are positive but because you didn't have enough counseling, you will not seek early care.”* (National policymaker #04)

This was especially a concern in the context of the *Treat All* national policy with the focus on linking all HIV positive cases to care and treatment upon an HIV diagnosis. Some national policymakers specifically perceived HIVST to be a hindrance to the implementation of the national policy agenda of *Treat All*.

“*The ‘treat all policy' will be affected, in that we want to put all infected persons on treatment, and in this case, people who test positive and not availing themselves will not be on treatment, so to some extent it's affecting that policy”* (National policymaker #01)

One national level policymaker mentioned the importance of supporting KP-friendly drop-in centers to facilitate KPs to seek services as KPs are more likely to attend KP-friendly drop-in centers as opposed to mainstream health facilities.

“*I wish to recommend that because the drop-in centers are … working for key population and vulnerable population, they feel very comfortable to access services there so why not strengthen the system and if it becomes I should say the Ghana Health Service should rather support the DICs [drop-in centers] and the implementation of the DICs and take over from partners and ensure that it is well resourced and bring in the necessary doctors to provide services for the KPs.”*

#### Facilitator 1: Increased Testing Modality That Would Increase Testing Uptake

The most common individual-level facilitator for the introduction of HIVST in the country mentioned by policymakers was that HIVST would be an additional strategy to complement all other existing testing strategies and would increase overall HIV testing rates. The current standard protocol for HIV testing in Ghana includes facility-based testing and community-based testing (e.g., outreach, door-to-door), both of which are performed by the provider. Many believed that the introduction of HIVST would strengthen the HIV testing program by making more testing options available to the beneficiary population and consequently increase HIV testing rates. Some policymakers confirmed that HIVST will offer testing options and not replace the existing testing strategies:

“*It* [HIVST] *will aid in increasing the number of people who will be willing to test to know their HIV status, … Yes, and the number of people who will be willing to test and also know their status will increase.”* (National policymaker #01)

“*I think it will just complement what is there already. It is not going to take away anything from the testing we already have.”* (Regional policymaker GA #7)

Many policymakers pointed to multiple reasons why they believed more people would test for HIV with the availability of HIVST, including increased privacy and confidentiality, reduced stigma, convenience, and less invasive.

“*The second benefit is associated with the stigma being associated with HIV. If the self-testing is being introduced, it will help people to do their own testing that would prevent them from going through the fear of stigma.”* (National policymaker #02)

“*One challenge is confidentiality issues and one is going to go through orientation and do it on her own or, something like that. On her own or on his own, he knows that confidentiality is ok, nobody knows the results.”* (National policymaker #01)

A few also mentioned that a great benefit of self-testing was the right of the individual to test and know his/her HIV status.

“*…to me it gives the person the right to decide … he or she can decide for his or her self when, how, under which conditions he or she should test his or her self for HIV.”* (Regional policymaker GA #07)

### Structural-Level Challenges and Facilitators

#### Challenge 1: Lack of a National Policy and Implementation Guidelines on HIVST

Among the structural level barriers raised by many policymakers was the absence of national guidelines on HIVST implementation in Ghana. The only available national level reference at the time of this study was the National Strategic Plan (NSP) 2016-2020 for HIV, which only recommended piloting HIVST among MSM, with no clear policy guidelines or framework on HIVST implementation in the country. The lack of national guidelines on HIVST in the country was identified as an obstacle to any successful introduction on HIVST in the country by some policymakers. Policymakers indicated that such policies would serve as a call to action and provide an operational framework for the rollout of HIVST.

“*Just because we don't have the policy in place is a barrier in itself. So maybe because we haven't made a point to bring it in the system, all those things rather become barriers.”* (Regional policymaker GA #07)

“*If we are going to roll it in the general population, something that we need to look out for … we have to integrate in a strategic plan, integrate it in our working document and also put it across for stakeholders to know the importance.”* (National policymaker #03)

One regional level policymaker mentioned that the current national guidelines and protocol for HIV testing in the country required that all HIV test must be supervised. The participant indicated that this may hinder effective implementation of HIVST without policy change.

“*Our current strategy is for you to freely go in and ask for the supervised testing that is all the policy now, meaning somebody has to administer it to you.”* (Regional policymaker GA #07)

#### Challenge 2: Cost of HIVST Kits

Many policymakers discussed the cost of HIVT kits and its implication for the national program as well as for the end users. At the national level, policymakers' concerns centered around the cost of procurement of the kits. Resources to finance HIVST programming was a concern to some policymaker as they are already strapped for financial resources to support antiretroviral drugs and services.

“*Short term, please we cannot (finance HIVST). We are struggling with ARVs for children even EID [early infant diagnosis] blood spots, we cannot cover them … so what we are talking about will re-channel the funds to self-testing — they will not do it.”* (National policymaker #03)

“*Policy barriers usually is the cost of the test kit because if it is going to be expensive then policy is not necessary”* (National policymaker #04)

However, others expressed that it should be a priority in the government programming and budgeting, particularly if it is part of the existing national HIV response and part of the national strategic plan. Some mentioned that it would be helpful if donors and other entities could help finance HIVST programming until it becomes part of the national program. “*Short term, if it's in the government policy, then it can be pre-financed. But if we can advocate to other individuals [donors], NGOs to help pre-finance until it comes to stay, then maybe the government can accept it.”* (Regional policymaker GA #07)

For the end users, if it should be made available through retail distribution outlets (e.g., private pharmacies, supermarkets), it may not be affordable to the consumers. Some policymakers expressed that with facility and provider HIV testing being free, the cost of self-test kit to beneficiary population will become a hindrance to uptake of testing.

“*And then other thing we know is this test is quite expensive. So, how affordable will it be for people to use it for self-test? Are they going to buy at the pharmacy? What is the cost going to be? So, maybe for those who really need to do the test, the cost may be far above what they can afford. But in the health facility it is free but if it is self-testing, it means you have to go and buy it…. So, the richer, they will be able to buy it and afford it and use. But most of the people who are infected I will say still are the people who are poor. So, if you want to reduce the HIV transmission you should think about the cost of the test kit”* (National policymaker #04)

One of the regional policymakers indicated that while there would be high acceptability among beneficiary population, the cost of the test kit will be a potential barrier, and there should be a modality of cost removal at the beginning and cost-share or total cost transfer to end users later.

“*The acceptability, how they will accept it, it is very important that they know and of course the cost involved. … Depending upon how they embrace it, then later on, they can even bring in the idea of either cost sharing* [subsidies] *or either to buy* [full cost recovery]*.”* (Regional policymaker BA #03)

#### Challenge 3: Supply Chain Management of HIVST Commodities

Many policymakers felt that the management of the supply chain would be critical to ensuring commodity security. Almost all national and a few regional policymakers indicated that without effective supply chain and logistics management systems in place, it would be a challenge to ensure commodity security and HIVST stock availability.

“*Shortage, wrong distribution and sometimes poor management of stock.I understand in some cases we have expired commodities in some of the facilities, all because of poor management and they are all gaps. Meanwhile at a particular stage, some partners are ready to receive from a particular channel and yet they don't get.”* (National policymaker #01)

Some policymakers indicated that the current challenges confronting commodity security (e.g., stocks, storage, and handling) could also affect the quality and integrity of the HIVST kits.

“*It also another thing even the storage of the test kits. Ordinary test kits we are facing challenges with storage and how much more bringing in self-test kits and how we even channel it to sell instead of giving it out free.”* (National policymaker #03)

“*Safety in handling the commodities could also be affected because … it's a form of chemical. If it is not kept at the right temperature or handling well, its safety can be affected.” (Regional policymaker BA #04)*

To ensure accountability, some policymakers called for the establishment of appropriate monitoring systems. They expressed the need for policy guidelines, structures, and systems to support effective monitoring of test kits to track not only the distribution, but also the usage of the kits. “*… I am talking logistical management system and policies on how to track and monitor, policy on the usage on the test kits, policy on reporting. We need to get how the structures should be. We need to also know there is a plan for system and ability of a program.”* (National policymaker #3).

#### Challenge 4: Data Monitoring and Reporting of Positive Cases

Some policymakers mentioned the negative impact that HIVST implementation would have on the national HIV programming and planning, particularly regarding data monitoring and reporting. Accurate data is pivotal for planning and monitoring any program, and many wondered how data would be assembled and fed into the national database. Several policymakers highlighted the need for having a system in place to report positive cases resulting from HIVST. It was particularly worrisome for them that under HIVST, positive cases may be missed in the reporting system if the HIVST user does not return to the health facility for confirmatory testing, where the person would be captured into the health information system.

“*I think, first implication is data management because at the moment, I don't see how those who will be tested through this system would be captured in our data. In the first place, it is because of stigma and other things that the person went and tested. So how are we able to tell that, let's say, we have 20 people who have tested … we should also know out of the number tested, how many positive and others are, but that limitation is going to be there.”* (Regional policymaker BA #02)

“*It is only those who will go to the health centers for confirmation that probably they can record there. So, where we don't have them coming to record or to show themselves, then data is going to be wrong.”* (National policymaker #01)

#### Facilitator 1: Reduced Burden on Health System

One of the greatest benefits many policymakers saw with the introduction of HIVST was that HIVST would reduce the burden on the health system as a result of reduced client load at the health facilities. With HIVST, only reactive cases would seek confirmation at the facilities, thus reducing the burden on the overly stretched and under-staffed health facilities. The resultant will be an improvement and efficient service delivery, and staff would be task-shifted to provide other essential health services.

“*I think when it comes to the area of testing, staffing, at that area will come down…Because a lot of people might not need to come* [to the health facility]. *If already the person is negative, that person will not come to the facility.”* (Regional policymaker BA #04)

#### Facilitator 2: Contribute to Achieving Testing Targets

Another structural level facilitator for the introduction of HIVST that was mentioned by a few policymakers was its potential to help meet HIV testing targets and help reach the global 90-90-90 targets for HIV epidemic control.

“*If we have the available test kits for self-testing and we have the funding for its implementation among KPs, I will be part of number one people to support the initiative. … because we are trying to achieve 90-90 objective by 2020. … if Ghana wants to be part of those who have been able to take up the sustainable development goal, then we need to start now and ensure that by 2020, we have been able to identify all those living with HIV within the community who do not know their status…and can be put on treatment so that we avert death.”* (National policymaker #04)

### Policymakers Support for HIVST

Despite the many challenges expressed, the majority of policymakers were highly in favor of the introduction of HIVST in Ghana, particularly for KPs.

“*If we have the available test kits for self-testing and we have the funding [for] its implementation among KPs, I will be part of number one people to support the initiative.”* (National policymaker #03)

However, there were divergent positions regarding the timing of the initiative. The support for immediate introduction centered around two main issues previously mentioned: increased testing uptake allowing more people to know their status and the potential to meet testing targets. A few policymakers, however, were more cautious in recommending immediate roll-out of HIVST. They felt that public sensitization, capacity building around HIVST of both implementors and end users, and development of an appropriate monitoring system were needed prior to the introduction of HIVST widely.

“*I would not support it until capacities have been built. So, I would look at later stage because I am of the view that until all these things we have mentioned are in place [monitoring system, sensitization], we wouldn't be able to have a very effective system. So not now as in today but at least we need some preparations.”* (Regional policymaker BA #02)

“*I will say later [introduction of HIVST] because if a lot of education doesn't go into it, it could be introduced and people might not patronize.”* (Regional policymaker BA #04)

## Discussion

The focus of this study was to gain in-depth understanding of policymakers' perspectives on the barriers and facilitators to the introduction of HIVST as a national HIV strategy. While many studies have reported on high acceptability and usage of HIVST, only a few have reported on the perspectives of policymakers (32, 33), which is critical to the successful large-scale implementation of HIVST. This study revealed that while policymakers were supportive of HIVST implementation in Ghana and that there was no doubt that HIVST would help increase HIV testing uptake, there were a number of client level as well as structural level issues that needed to be addressed before large-scale implementation.

One of the greatest individual-level concerns of policymakers was the absence of counseling and the consequences of lack of psychosocial support, and the counseling around the need for confirmatory testing and linkage to care and treatment for HIVST users who obtain a reactive test result. This is a well-documented concern about HIVST among key stakeholders including healthcare providers, policymakers, academics, activists, donors, among other, in other African countries (34). This concern is certainly warranted; in fact, a recent meta-analysis found that while HIVST significantly increased uptake, linkage to care and treatment was lower compared to standard HIV testing (35). When HIVST occurs in a supervised manner (i.e., aided by a healthcare provider), psychosocial support, counseling, and linkage to care, prevention, and treatment services can be facilitated by leveraging existing HTC services (10). However, for unsupervised HIVST, strategies are needed to provide counseling and facilitate linkage to care. At a minimum, test kits should contain key counseling messages including information on the need for confirmatory testing following an initial reactive self-test using both written (local language) and pictorial instructions. However, to conduct more active follow-up, obtaining contact information and unique personal identifiers (including biometrics) of clients is key to facilitate counseling text-messaging (along with specific locations of HIV clinics) and phone-based follow-up (10). In Ghana, a few strategies using community-based platforms have been piloted with success among the MSM population (36). These pilot interventions showed that virtual community-based platforms through mobile and digital technology could be used to link MSM to HIV care providers. One intervention provided access to peers *via* an online app for peer support and referral to the providers (36). Toll-free telephone hotlines, online counseling, and automated text messaging may also be considered for counseling and facilitating linkage to care (8, 10, 19, 37–40). A study among Nigerian MSM to whom HIVST kits were distributed found that while a hotline was available to study participants, it was rarely used; rather, participants preferred to contact the peer educators from whom they received the HIVST kits as they preferred to go to a known trusted source rather than an anonymous hotline (19). A survey among a representative sample of potential HIVST users were asked about their intention in linkage to care and their preferences for strategies (41). Eighty-five percent indicated they would link to care within the first week of a positive test result and home visits (53%) were preferred over a phone call (30%) or SMS (17%) to be reminded to be linked to care. Currently, there is limited evidence on what strategies are effective in linking self-testers to care and treatment. Selection of the mode of follow-up for counseling and linkage to care and treatment is very context and population -specific; therefore, implementers will need assess what will work in their context given the available resources. While policymakers in this study mentioned their concerns only around linkage to care and treatment for those who test positive with HIVST, linkage to prevention services, including PrEP, is also important for those who test negative (42). HIVST may be a promising approach to increase linkage of high-risk populations to PrEP and subsequently increase PrEP usage.

Policymakers in this study pointed out the paramount nature of the need for the inclusion of HIVST as a programmatic approach in the NSP and country-specific HIVST implementation guidelines and policy framework/directive in order to be able to implement HIVST in the country. This is consistent with other studies (7, 24, 32, 33, 43–46) that attest that HIVST will require the institutionalization of national implementation guidelines pivoted on outcomes of implementation science research in order to have nationwide large-scale roll-out. At the time of the study, the NSP 2016-2020 had only mentioned the need to pilot HIVST in Ghana before a policy directive could be made. After the completion of this study, national and regional level stakeholder technical discussions were held and Ghana Health Service/National AIDS Control Program led the development of the HIVST guidelines. Additionally, HIVST has been added to the draft of the 2021-2025 National Strategic Plan as a strategy, especially to improve testing for KPs and adolescent girls and young women. While this will make large scale roll-out of HIVST easier, barriers mentioned by the policymakers in this study are still relevant given challenges in nascent stages of real-world introduction of new strategies (as opposed to a study or pilot setting).

The newly-drafted Ghana HIVST implementation guidelines addresses the need for the integration of HIVST kits into the country's supply chain management system to ensure appropriate quantification, distribution and inventory management, product quality assurance, and data reporting. It is envisioned that as the country transitions from a manual data reporting system to an electronic system (Ghana Integrated Logistics Management Information System), there will be more efficient tracking of stock availability and usage of kits from service delivery points, thereby ensuring a more effective supply chain management system.

Direct cost of HIVST kits to the individual constitutes a significant barrier to wider adoption, access and utilization (47–50). If HIVST should be made available through pharmacies and other retail outlets, the price of the kits must be affordable to ensure equitable access. Willingness-to-pay studies in Cote d'Ivoire, Tanzania, and Kenya have shown that people are willing to pay USD 0.87 (Tanzania) to USD 1.77 (Cote d'Ivoire) (51, 52). Partial or full subsidization may need to be considered for low-income populations to increase access and coverage (51, 52). Lastly, public–private partnerships should be considered as an option to facilitate transition to domestic country budgets as donor funding for HIVST programs decrease (53). The cost-effectiveness of HIVST hinges on the benefits of early diagnosis leading to improved treatment outcomes; however, this is dependent on high prevalence of undiagnosed HIV (54). Therefore, a targeted approach to HIVST distribution will be key to ensuring a more cost-effective approach. As mentioned in the new NSP 2021-2025 and the HIVST implementation guidelines, the main target populations for HIVST must be KPs as it will have the greatest impact.

Issues on data capturing and monitoring for programming and assessing the level of coverage were also mentioned as technical areas that needed to be addressed before the large-scale implementation of HIVST. Potential strategies to address this challenge include the use of internet and interactive text message surveys to follow-up with HIVST clients regarding their usage and result of the test (10, 42, 55). This will not only capture HIVST usage and results but could allow for automated referral to post-test services. An important issue to address with these solutions is the need to ensure confidentiality of clients when reporting their self-test results. Routine surveillance surveys such as bio-behavioral surveillance surveys and AIDS Indicator Surveys should also include questions about HIVST usage to determine population coverage of self-testing as well as successful linkage to prevention and treatment (10, 42, 55).

One of the greatest structural level appeals of HIVST as mentioned by many providers was the potential reduced burden on the health system. Only those who self-test positive will need to come in for confirmatory testing to be conducted by a provider; those who test negative can be referred for preventive services such as PrEP. This helps to reduce the burden and time of HIV testing on healthcare providers, thereby improving the efficiency and effectiveness of the health system (42), which will be even more critical as HIVST expands.

A limitation of this study was that regional level policymakers were selected from only two region (out of 16 regions) and thus their views may not be reflective of views held by policymakers from other regions of Ghana. However, national level policymakers are tuned into regional level issues around HIV programming, policies, and implementation issues, and hence, their perspectives likely also convey those of regional policymakers.

## Conclusion

Overall, this study revealed that while many stakeholders see the added value of HIVST for epidemic control, the roll-out of HIVST must be preceded with policy framework and implementation guidelines, education and sensitization of the population and systems in place to address the various client and structural level challenges. The concerns raised related to counseling, linkage to HIV prevention, care, and treatment, supply chain challenges, monitoring and reporting, and the costs of HIVST kits should be critically considered and addressed. National guidelines on HIVST will support existing HIV policies and strategies and position HIVST as an important complement to existing HTS strategies in Ghana.

## Data Availability Statement

The raw data supporting the conclusions of this article will be made available by the authors, without undue reservation.

## Ethics Statement

The studies involving human participants were reviewed and approved by Population Council Institutional Review Board, New York (USA) and Ghana Health Service Ethical Review Committee, Accra. The patients/participants provided their written informed consent to participate in this study.

## Author Contributions

HN: research design, data interpretation, paper writing, and revision. EA and HT: research design, protocol development, data collection training and supervision, analysis, paper writing, and revision. WT: research design, protocol development, data collection training, analysis, paper writing, and revision. AA: paper review and revision. YR and MK: research design, paper review. SA, KA, and EE: research design, protocol development data interpretation. All authors contributed to the article and approved the submitted version.

## Funding

Funding support was from the USAID under the USAID Strengthening the Care Continuum Project, Cooperative Agreement Number AID-641-A-16-00007.

## Conflict of Interest

The authors declare that the research was conducted in the absence of any commercial or financial relationships that could be construed as a potential conflict of interest.

## Publisher's Note

All claims expressed in this article are solely those of the authors and do not necessarily represent those of their affiliated organizations, or those of the publisher, the editors and the reviewers. Any product that may be evaluated in this article, or claim that may be made by its manufacturer, is not guaranteed or endorsed by the publisher.
